# O-GlcNAc: A Sweetheart of the Cell Cycle and DNA Damage Response

**DOI:** 10.3389/fendo.2018.00415

**Published:** 2018-07-30

**Authors:** Caifei Liu, Jing Li

**Affiliations:** Beijing Key Laboratory of DNA Damage Response and College of Life Sciences, Capital Normal University, Beijing, China

**Keywords:** O-GlcNAc, mitosis, replication, cytokinesis, DNA damage response

## Abstract

The addition and removal of O-linked N-acetylglucosamine (O-GlcNAc) to and from the Ser and Thr residues of proteins is an emerging post-translational modification. Unlike phosphorylation, which requires a legion of kinases and phosphatases, O-GlcNAc is catalyzed by the sole enzyme in mammals, O-GlcNAc transferase (OGT), and reversed by the sole enzyme, O-GlcNAcase (OGA). With the advent of new technologies, identification of O-GlcNAcylated proteins, followed by pinpointing the modified residues and understanding the underlying molecular function of the modification has become the very heart of the O-GlcNAc biology. O-GlcNAc plays a multifaceted role during the unperturbed cell cycle, including regulating DNA replication, mitosis, and cytokinesis. When the cell cycle is challenged by DNA damage stresses, O-GlcNAc also protects genome integrity via modifying an array of histones, kinases as well as scaffold proteins. Here we will focus on both cell cycle progression and the DNA damage response, summarize what we have learned about the role of O-GlcNAc in these processes and envision a sweeter research future.

## Introduction

The study of O-linked N-acetylglucosamine (O-GlcNAc), O-GlcNAc transferase (OGT), and O-GlcNAcase (OGA) was pioneer by Dr. Hart in 1984 (1). OGT modifies the substrate protein at Ser/Thr residues with the O-GlcNAc group, while OGA reverses it. Since then, both biologists and chemists have been working hand in hand to solve the sweet mystery. In this review, we will first cover the laboratory routine to study protein O-GlcNAcylation, and then venture onto the recently identified function of O-GlcNAc in regulating cell cycle and the DNA damage response (DDR). For the versatile role of O-GlcNAc in other biological processes, please refer to other comprehensive and exhaustive reviews (2–5).

## An overview of O-GlcNAc

Of all the glucose that we consume every day, ~2−5% enters the hexosamine biosynthetic pathway (HBP), which provides UDP-N-acetyl-D-glucosamine (UDP-GlcNAc) (6), the donor substrate for OGT. UDP-GlcNAc is highly responsive to cellular nutrient variations, as its synthesis relies heavily on the metabolism of glucose, amino acids, fatty acids, and nucleotides (7). Hence, O-GlcNAc may serve as a reporter for the functional status of multiple pathways and is considered an ideal metabolic sensor (8), and defunct O-GlcNAc signaling underscores many metabolic diseases (3, 5).

### O-GlcNAc is implicated in various human diseases

O-GlcNAcylation is most abundant in the pancreas, followed by the brain (3). Thus it has been intimately linked with a plethora of human diseases, especially diabetes, and Alzheimer's disease (AD).

In diabetes, hyperglycemia leads to chronic hyper-O-GlcNAcylation, which brings mayhem to cellular signaling networks. And inhibiting O-GlcNAc was found to blockade arrhythmias in diabetic animals (9). Recent investigations show that OGT in pancreatic β cells regulates the β cell mass. And OGT disruption results in diabetes via the endoplasmic reticulum (ER) stress and the Akt pathway (10). The vital player in AD, Tau, is O-GlcNAcylated in normal brains, but hyperphosphorylated in AD brains (3). Interestingly, increasing O-GlcNAc in mice decreases neuronal losses (11).

### O-GlcNAc in cancer metabolism

One characteristic of cancer cells is the Warburg effect, namely, elevated glycolytic flux, including glycolysis, the pentose phosphate pathway (PPP), and the HBP. Moreover, enhanced O-GlcNAcylation levels have been pinpointed as a common cancer feature (12). Many studies have impinged O-GlcNAc as a sweet accomplice of cancer.

Upon entering the cell, glucose is phosphorylated by hexokinase to become glucose-6-phosphate (G6P). G6P then either undergoes glycolysis and the tricarboxylic acid (TCA) cycle to generate ATP, NADPH, and pyruvate, or produce ribose-5-phosphate and NADPH through PPP (13). PPP maintains redox homoeostasis in rapidly dividing cells, especially in cancer cells. In the glycolytic pathway, phosphofructokinase 1 (PFK1) is O-GlcNAcylated at S529 upon hypoxia, inhibiting PFK1 activity and directing the glucose metabolism to the PPP (14). Concomitantly, the rate-limiting enzyme of the PPP, G6P dehydrogenase (G6PD), is also O-GlcNAcylated during hypoxia, thus being activated to increase glucose flux via PPP (15). Taken together, O-GlcNAc activates the PPP to promote cancer proliferation.

In the TCA cycle, fumarase both reversibly catalyzes fumarate to malate in the mitochondria, and regulates amino acid and fumarate metabolism in the cytoplasm. Fumarase is O-GlcNAcylated at S75, which antagonizes phosphorylation by AMPK at the exact same residue when glucose is scarce (16). pS75 mediates fumarase-ATF2 interaction, blocks KDM2A activity, stabilizes H3K36me2 and thus redirecting the cell proliferation to growth arrest (16). Hence in cancer cells, where OGT activity is particularly high, pS75 levels are relatively low, conferring growth advantage to cancer cells.

Equally important, OGT also regulates lipid metabolism in cancer. To sustain growth, cancer cells usually utilize *de novo* lipogenesis, which encompass activation of key enzymes, such as fatty acid synthase (FAS), and master transcription factors, such as the sterol regulatory element binding protein (SREBP-1). OGT suppression leads to lipogenic defects, which could be rescued by SREBP-1 overproduction (17). OGT regulates SREBP-1 protein abundance, probably via AMPK (17). On the other hand, FAS binds with OGA, and the interaction increases during oxidative stress (18). FAS inhibits OGA activity, so the O-GlcNAc levels elevate under oxidative stress in mammalian cells (18).

In conclusion, O-GlcNAc integrates various nutrient signaling with growth signaling and may provide new venues for therapeutic purposes.

### Crosstalks between O-GlcNAc and other post-translational modifications (PTMs)

Then how does O-GlcNAc weave its magic wand? The answer lies largely in crosstalk with other PTMs. Due to the large size of the O-GlcNAc moiety, steric hindrance is imposed upon the O-GlcNAcylated protein, thus impeding other PTMs at the same or adjoining sites. For instance, when a mixture of two OGA inhibitors, PUGNAc and NAG-thiazoline, was utilized to analyze the ~700 phosphopeptides, phosphorylation levels of 131 peptides (18.4%) escalated and 234 (32.9%) peptides dampened (19). In a quantitative phosphoproteomics study using OGT wild-type and null cells, 232 phosphosites increased and 133 decreased out of the 5,529 sites in the null cells (20). Thus, a yin-yang relationship has been proposed between O-GlcNAcylation and phosphorylation (3).

Discordant results against the “yin-yang” model have also been recorded. In a 2008 study, researchers closely monitored phosphorylation sites when O-GlcNAcylation was elevated (19). As a result, 280 phosphorylation sites decreased, while 148 sites increased (19). In a recent quantitative phosphoproteomics study, a great many DDR proteins were identified to be O-GlcNAcylated, among which was checkpoint kinase 1 (Chk1) (20). When OGT is deleted, pT113 of Chk1 increases, consistent with the “yin-yang” model, but pS151 decreases, against the model (20). Another case in hand is the intermediate filament protein, vimentin. Vimentin filament severing is a key step for completion of cytokinesis, and many phosphorylation events intricately mediate this process. In particular, cyclin-dependent kinase 1 (CDK1) phosphorylates vimentin at S55 (21) to prime vimentin for subsequent phosphorylation by polo-like kinase 1 (Plk1) at S82 (22), thus inhibiting vimentin filament assembly. Other kinases, such as Aurora B and the Rho kinase, also phosphorylate vimentin at S72 and S71, respectively, thus localizing vimentin to the cleavage furrow (23–25). When cells were depleted of OGT by siRNA, pS71 levels were hampered; vimentin filament thus could not be severed during cytokinesis, leading to cytokinesis failure (26). In sum, the relationship between phosphorylation and O-GlcNAcylation needs to be analyzed case by case, and there might not be a clear-cut rule.

The relationship between O-GlcNAcylation and ubiquitination and hence protein stability was tested recently. Using a newly developed quantitative time-resolved O-linked GlcNAc proteomics (qTOP), 533 O-GlcNAcylated proteins were examined for stability, and 14% were identified to be hyper-stably O-GlcNAcylated (27). Of this pool of ~75 proteins, O-GlcNAcylation has a significant impact on the protein stability, and O-GlcNAcylation mainly promotes protein stability (27).

Congruent with this study, O-GlcNAcylation has been identified to augment protein abundance in an array of studies. O-GlcNAcylation of the circadian clock proteins, BMAL1 and CLOCK, inhibits their ubiquitination and stabilizes protein levels (28). During gluconeogenesis, the master regulator PGC-1α is O-GlcNAcylated to bind the deubiquitinase BAP1, thus dampening ubiquitination, and enhancing protein abundance (29). The mixed lineage leukemia 5 (MLL5) protein forms a stable complex with OGT and ubiquitin specific protease 7 (USP7), and OGT suppresses MLL5 ubiquitination and increases its stability (30). The histone methyltransferase enhancer of zeste homolog 2 (EZH2) is O-GlcNAcylated at S75, which maintains its protein stability (31). Besides proteasome-mediated degradation, ubiquitination also plays other roles in signal transduction. And crosstalks have also been identified in these scenarios. For instance, O-GlcNAcylation of DNA polymerase Polη promotes its polyubiquitination and subsequent removal from replication forks (32).

With more people joining in the O-GlcNAc venture, communication among O-GlcNAc and more PTMs, such as methylation, SUMOylation, acetylation, ADP-ribosylation is bound to be unveiled.

## The toolbox of O-GlcNAc research

As the Chinese saying goes, “Nice craftsmanship entails utilization of nice tools.” The toolbox of O-GlcNAc has been limited, compared to other PTMs, e. g., phosphorylation and ubiquitination. The common practice to identify O-GlcNAcylated proteins is by immunoprecipitating (IP) proteins of interest, then immunoblotting (IB) with O-GlcNAc antibodies. Below we will briefly delineate the lab routine to identify and study protein O-GlcNAcylation.

### Antibodies

O-GlcNAc antibodies encompass RL2 and CTD110.6, among others (33). CTD110.6, an IgM, recognizes YSPTS(O-GlcNAc)PSK and also non-specifically binds terminal β-linked-GlcNAc (β-GlcNAc) and other N-glycan cores (34). RL2, on the contrary, is an IgG, and its antigen is pore complex-lamina fraction purified from rat liver nuclear envelopes (34). The RL2 and CTD110.6 are considered pan-O-GlcNAc antibodies, and might be promiscuous. They have overlapping, yet somewhat distinct ranges of protein targets. Hence it has been recommended to adopt both antibodies to conclude the O-GlcNAc modification. Alternatively, GlcNAc could be added during antibody blotting to compete against antibody binding, so that signals from O-GlcNAcylated proteins could be validated (35).

Another method is to perform *in vitro* O-GlcNAcylation assays. Using tritiated UDP-Galactose (UDP-[^3^H]-Galactose) as a donor, the addition of “hot” O-GlcNAc onto proteins can be traced. However, this is not a trivial experiment. First, the OGT enzyme is sensitive to salt and reducing agents, thus the purified proteins need to be desalted (36). Second, tritium has a long half-life of 12.3 years, so the storage of residue materials is a valid concern. Third, tritium is not as sensitive as ^32^P, hence autoradiography might take an extended period of time to be detected. Alternatively, RL2 and CTD110.6 could be used in IB experiments on the *in vitro* reaction products (37), thus avoiding the tritium issue.

### Inhibitors

The aforementioned identification of O-GlcNAcylated proteins could be facilitated by using OGA inhibitors, thus enhancing O-GlcNAc signals (36). Two most commonly utilized OGA inhibitors are O-(2-acetamido-2-deoxy-D-glucopyranosylidene) amino N-phenylcarbamate (PUGNAc) and Thiamet-G (TMG). PUGNAc is a 1,5-hydroximolactone. It is a non-selective inhibitor of OGA and inhibits glycosyl hydrolases in general, in particular lysosomal hexosaminidases (38). Treating 3T3-L1 adipocytes with PUGNAc increased globular O-GlcNAc levels and resulted in insulin resistance, the hallmark of diabetes (39), consistent with the current view that elevated O-GlcNAcylation correlates with diabetes. However, such effects could not be repeated by treating cells or animals with TMG (40). Since TMG is more specific for OGA compared to PUGNAc, the results above suggest that the effect of PUGNAc was non-specific. It is more promising in treating AD, as treating AD mice with TMG increased Tau O-GlcNAcylation, attenuated Tau phosphorylation and hence aggregation, thus opening new venues for AD therapy (11).

Unlike OGA inhibitors, OGT inhibitors have been difficult to come along (38). Alloxan is the first reported inhibitor for OGT (41). As a uracil analog, it inhibits OGT, but its toxicity affects many cellular processes (41). Other inhibitors have been used anecdotally. 6-diazo-5-oxo-L-norleucine (DON), which is an inhibitor of glutamine-utilizing enzymes, such as the CTP synthase (CTPS) and NAD synthase, has been shown to inhibit O-GlcNAcylation (35). Two OGT inhibitors have been reported recently. One is a naturally produced OGT inhibitor, L01 (42), whose effects are yet to be tested. The other is uridine diphospho-5-thio-N-acetylglucosamine (UDP-5SGlcNAc), a substrate analog of O-GlcNAc that might be a competitive inhibitor of OGT (43). When the inhibitor per-O-acetylated 2-acetamido-2-deoxy-5-thioglucopyranose (Ac-5SGlcNAc) is added into cells, it is coverted to UDP-5S-GlcNAc, which is the most frequently used OGT inhibitor today (44–46).

### Mass spectrometry (MS)

Last three decades have witnessed significant strides in developing MS instruments, especially electron transfer dissociation (ETD) MS (47, 48) and higher energy collisional dissociation (HCD) MS (49). These two apparatuses differ greatly from each other: HCD cannot specify the amino acid that is modified, while ETD is only efficient toward peptides. For more details about identification of O-GlcNAc sites by MS, please refer to a more in-depth review (33).

### Mutagenesis studies

Following MS, the potential sites will be mutagenized to confirm whether they are indeed the modification sites. O-GlcNAc-deficient mutations are normally Ser/Thr to Ala, but O-GlcNAc-mimicking mutations are not well defined. Wang et al. (50) mutated the O-GlcNAc sites of the Ser/Thr kinase AKT1, T305, and T312, to Tyr, to mimic the bulky steric hindrance imposed by the O-GlcNAc moiety. But it has not been commonly adopted.

The mutant proteins will subsequently be IBed against the O-GlcNAc antibodies, RL2 and CTD110.6. Site-specific O-GlcNAc antibodies are yet to be developed, unlike site-specific phospho-antibodies, which have been a standard practice to study protein phosphorylation.

Most of the time, MS identifies more than one O-GlcNAc site (32, 51, 52). It has been noted that in this scenario, single mutants will sometimes display elevated O-GlcNAcylation, while the double/triple/quadruple mutants will down-regulate O-GlcNAcylation (32, 51, 52). The single residue might be a preferred modification site, the abolishing of which might result in prolonged interaction between OGT and the target protein. But the exact molecular details are lacking.

## O-GlcNAc regulates cell cycle progression

The cell cycle, comprising G1, S, G2, and M phases, has entranced biologists from the earliest times. How is the genomic DNA faithfully replicated? How is the chromosome segregation process synchronized? How do the two daughter cells part from each other? These are just a few questions that have baffled biologists and the answers are still much sought after.

As has long been appreciated, the faithful execution and success completion of cell cycle is governed by a multitude of master kinases, phosphatases, ubiquitin E3 ligases and a network of protein machineries. Recent years have witnessed a surge of reports on the role of OGT in the cell cycle. Henthforth, we will first have a bird's eye view on the role of OGT, and then delve into the substrates of O-GlcNAc in cell cycle (Tables 1, 2). Of note, these tables are by no means comprehensive. Table 2 only contains proteins whose molecular mechanism is well studied. Many other proteins have been identified in many proteomic screens, but their functional significance is not well understood.

**Table 1:** Effects of OGT and OGA on cell cycle progression.

**Cell cycle phase**	**O-GlcNAcylation levels**	**OGT localization patterns, overproduction/deletion studies**	**OGA localization patterns, overproduction/deletion studies**
S phase	O-GlcNAc decreases (53)		OGA activity increases (53)
G2/M	Increased O-GlcNAc delays G2/M in cultured cells (54). Increased O-GlcNAc is observed in G2/M entry in *X. laevis* (55).		OGA inhibition hampered G2/M transition (56)
M phase	O-GlcNAc levels decrease (54, 57)	OGT localizes to the spindle (58).	OGA is ubiquitous, but absent from the newly formed nuclear membranes of the two daughter cells (54).
		OGT protein amounts decrease (58). OGT mRNA levels decrease (59) OGT overproduction leads to chromosome bridges, inhibits CDK1 activity (50), reduces both mRNA and protein levels of Polo-like kinase 1 (PLK1) (50).	OGA disruption results in lagging chromosomes and micronuclei (60); OGA knockdown HeLa cell lines manifested spindle defects and mitotic exit effects (61)
Cytokinesis		OGT localizes to the midbody (26, 57). OGT deletion results in vimentin bridges (26). OGT overexpression results in polypoidy (26, 54).	OGA is diffuse, not at the midbody (57). OGA disruption leads to cytokinesis failure (60)

**Table 2:** O-GlcNAcylated proteins during the unperturbed cell cycle^*^.

**Cell cycle phase**	**Protein name**	**Molecular details**	**References**
G1 phase	Retinoblastoma (Rb)	Rb is O-GlcNAcylated *in vitro* and *in vivo*. And this modification increases in G1.	(62)
	Minichromosome maintenance protein (MCM)	Mcm3,6,7 are O-GlcNAcylated.	(53)
M phase	Histone 3 (H3)	H3 is O-GlcNAcylated at T32, and antagonizes phosphorylation at S10 of H3.	(56)
		Increasing UDP-GlcNAc suppresses phosphorylation at S10 of H3.	(63)
	Cdh1	Cdh1 is O-GlcNAcylated in both cultured cells and mouse brain extracts. O-GlcNAc antagonizes its phosphorylation, promotes its interaction with Anaphase Promoting Complex/Cyclosome (APC/C) and enhances the activity of APC/C.	(51)
	NuMA1	O-GlcNAcylated NuMA1 interacts with Galectin-3, localizes to the spindle pole, essential for mitotic spindle cohesion	(64)
	Ewing Sarcoma Breakpoint Region 1 (EWS)	EWS is O-GlcNAcyated to promote its nuclear localization. O-GlcNAc of EWS increases in OGA KO cells, resulting in uneven distribution of the spindle mizdone	(65) (61)
Cytokinesis	Vimentin	Vimentin is O-GlcNAcylated. During cytokinesis, O-GlcNAcylation promotes phosphorylation of S71 of vimentin, results in vimentin filament disassembly and ensures a complete cytokinesis.	(57) (26, 66)

* *This table only contains proteins whose molecular mechanism is well studied. Many other proteins have been identified in many proteomic screens, but their functional significance is not well understood*.

### An overall role of OGT/OGA in the cell cycle

Although a late comer to the cell cycle arena, there has been mounting evidence that OGT underscores cellular proliferation (67).O-GlcNAc levels fluctuate during distinct phases of the cell cycle. Upon entering the S phase, the OGA activity increases and the global O-GlcNAc levels decrease (53). During G2/M, reports have been incongruous. In *X. laevis* oocytes, global increase of O-GlcNAc is discernable during G2/M transition (55). In cultured mammalian cells, however, increased O-GlcNAc delays G2/M transition (54), and OGA disruption hinders G2/M transition (56). The discrepancy could be partly due to different species.

During mitosis, O-GlcNAc levels decrease (54, 57). OGA disruption results in lagging chromosomes and micronuclei (60), and stable OGA knockdown HeLa cell lines manifest aberrant spindles and mitotic exit effects (61). Meanwhile, OGT overproduction leads to chromosome bridges and delays mitosis (50). The mitotic spindle is an important apparatus to ensure accurate chromosome segregation (68), and its integrity impinges on the appropriate level of O-GlcNAcylation, as either OGT or OGA overproduction leads to aberrant mitotic spindles (69).

During cytokinesis, both OGA disruption and OGT overexpression lead to polyploidy and cytokinesis failure (26, 54, 60). Studies from our lab show that Chk1 phosphorylates OGT at S20 specifically during cytokinesis (26). This modification stabilizes OGT abundance and ensures a sufficient level of pS71 of vimentin (26). Thus OGT knockdown leads to vimentin bridges (26). In sum it is suffice to say that an appropriate O-GlcNAc level is pivotal for all phases. If chemical interrogation or genetic ablation renders O-GlcNAc above or below that level, cellular reproduction will be seriously affected.

Cytologically, OGT localizes to the spindle during M phase (54, 58). And it localizes to the midbody during cytokinesis (26, 54, 57). The localization pattern of OGA is quite different. At mitosis, OGA is ubiquitous, but absent from the newly formed nuclear membranes of the two daughter cells (54). During cytokinesis, OGA is diffused, without a distinct midbody localization (57).

### O-GlcNAc and the mitotic master kinases are intertwined

A link between O-GlcNAc and the mitotic master kinases have also been identified. The cell cycle is regulated by a concerted choreography of cyclins and CDKs (70). Investigations show that O-GlcNAc modulates cyclin stability. To begin with, cyclin D abundance increases during G1 and declines in S and M phases. OGA overproduction delays the increase of cyclin D (54), while OGT overproduction constitutively decreases cyclin D levels, partly due to delayed mitosis (54). Another example is cyclins A and B. Cyclin B peaks during prophase and decreases during metaphase, and cyclin A peaks in G2. In cells that overproduce OGT or OGA, protein abundance of cyclins A and B fails to decline, probably due to mitotic exit defects (54).

**Figure 1:**
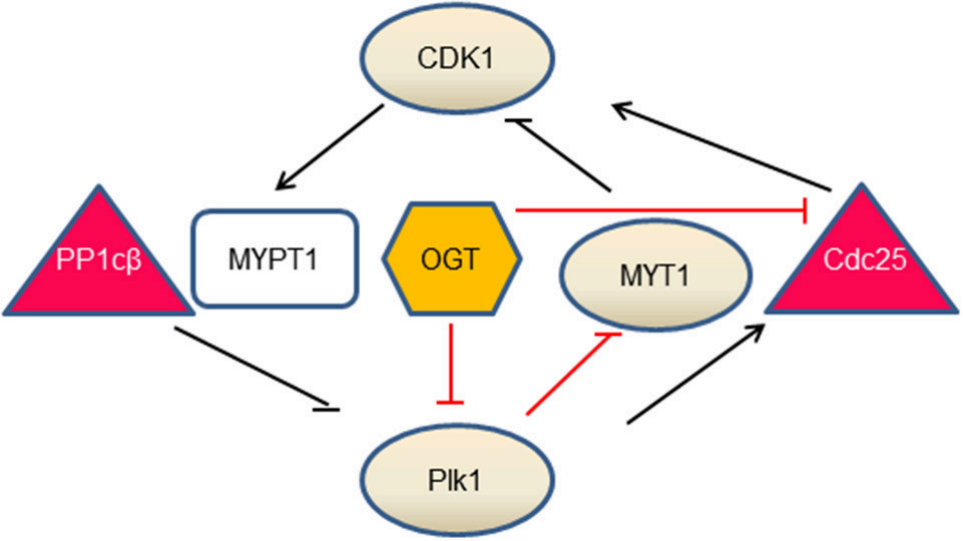
The interlinked network between O-GlcNAc and mitotic kinases. Our current understandings comprise three branches. During mitosis, CDK1 phosphorylates MYPT1 to promote its interaction with Plk1. MYPT1 recruits PP1cβ to dephosphorylate and inactivate Plk1 (71). In the second branch, OGT decreases Plk1 protein levels, which further increases MYT1 levels and decreases MYT1 phosphorylation. As MYT1 inhibits CDK1 by phosphorylation, OGT thus promotes inhibitory phosphorylation of CDK1. In the third branch, OGT inactivates CDK1, via down-regulating the mRNA levels of Cdc25-the activating phosphatase of CDK1 (50). Whether OGT is enmeshed in the MYPT1 branch is not understood. Kinases are in circles, phosphatases are in triangles, MYPT1 is in a square and OGT is in a hexagon. Lines in black denote direct effects (phosphorylation or dephosphorylation), and lines in red demarcate indirect effects (mRNA or protein levels).

O-GlcNAc regulates two key mitotic kinases, CDK1 and Plk1 (Figure 1). CDK1 regulates Plk1 through Myosin phosphatase targeting protein 1 (MYPT1), which is a targeting subunit of protein phosphatase 1cβ(PP1cβ) (72). During mitosis, Cdk1 phosphorylates MYPT1 at S473, creating a binding pocket between MYPT1 and Plk1. Thus MYPT1 recruits PP1cβ to dephosphorylate Plk1 at pT210, the activation phosphorylation site (71). Intriguingly, MYPT1 is also a targeting subunit of OGT in neuroblastoma cells (73), raising the tantalizing possibilty that MYPT1 targets both PP1β and OGT to the same substrate, so that the substrate protein could be dephosphorylated and O-GlcNAcylated simultaneously. Indeed, Plk1 is identified to be O-GlcNAcylated in an *in vitro* OGT assay (74), but whether this occurs *in vivo*, or O-GlcNAcylation interplays with pT210 of Plk1 is elusive. To add yet another layer of complexity, MYPT1 itself is O-GlcNAcylated (73). We are yet to find out the exact function of this PTM.

Overproduction of OGT decreases mRNA levels of Plk1 and therefore Plk1 protein levels, which further decreases the abundance of Membrane Associated Tyrosine and Threonine cdc2 inhibitory Kinase (MYT1) and increases the activity of MYT1. As MYT1 phosphorylates CDK1 in an inhibitory manner, thus OGT overexpression increases the inhibitory phosphorylation of CDK1 (50). Another branch of the network concerns Cdc25, the activating phosphatase of CDK1. On one hand, Plk1 phosphorylates Cdc25, which in turn dephosphorylates and activates CDK1. On the other hand, OGT overproduction curbs mRNA levels of Cdc25 and subsequent protein levels, thus inactivating CDK1 (50). In sum, O-GlcNAc exerts its role through protein abundance at the transcriptional level. OGT overproduction increases the inhibitory phosphorylation of CDK1, and decreases phosphorylation levels of its substrates (50).

A third key kinase, Aurora B, is also extensively studied (75). Aurora B is a component of the chromosomal passenger complex (CPC) that also comprises INCENP, Survivin, and Borealin. CPC is an essential driver of the late mitotic events, including assembly and disassembly of the mitotic spindle, activation of the spindle assembly checkpoint and cytokinesis (75). Both OGT and OGA are in a complex with Aurora B and protein phosphatase 1 (PP1) during M phase by coIP studies (57). Moreover, chemical inhibition of Aurora B enhances OGT protein abundance and therefore cellular O-GlcNAcylation levels during M phase (57). Importantly, the spindle localization of OGT is derailed by Aurora B inhibition (57). It will be of particular interest to investigate whether Aurora B phosphorylates OGT or OGA in due course.

### O-GlcNAcylated proteins in the S phase

The retinoblastoma protein (RB) is a classical tumor suppressor that is key for the replication process (76). In the G1 phase, Rb is hypophosphorylated and binds the transcription factor E2F-1, an essential transcription factor for many S phase genes. In the late G1 phase, Rb is hyperphosphorylated by CDKs, thus releasing E2F-1 to transcribe downstream S phase genes (76). Consequently, cells will enter S phase. Both *in vitro* and *in vivo* experiments show that Rb is O-GlcNAcylated, with the highest O-GlcNAc levels in the G1 phase, suggesting that O-GlcNAcylation of Rb antagonizes CDK-dependent phosphorylation and inactivates the transcriptional activities of E2F1 (62). It will be of further interest to pinpoint the modification residues and study its effects on cell cycle.

Conversely, the transcription levels of *OGT* and *OGA* are also regulated by Rb/E2F1 (77). *OGT* and *OGA* expression is reduced by overproduction of E2F1 in HEK293 cells and is increased in *E2F1*^−/−^ mouse fibroblasts and *Rb*^−/−^mouse embryonic fibroblasts (MEFs). E2F1 overproduction does not change *OGT* or *OGA* expression levels in *Rb*^−/−^MEFs, suggesting that E2F1 negatively regulates *OGT* and *OGA* expression in an Rb-dependent manner (77). Mechanistically, consensus E2F binding sequences were identified on *OGT* and *OGA* and confirmed by reporter-based assays and *in silico* modeling (77). Therefore, expression of *OGT* or *OGA* is not constant, but subject to multiple layers of regulations.

The minichromosome maintenance (MCM) replicative helicase, comprising Mcm2-7, is loaded onto the DNA in the G1 phase, thus restricting one round of DNA replication per cell cycle (78). The Mcm2-7 is activated in S phase to unwind DNA. Investigations have shown that loading and activation never occur concomitantly, and not all MCMs loaded are activated in one cell cycle (79). Based on the recent cryoelectron microscopy (cryo-EM) data, an elaborate Acrobat Model has been proposed to elucidate how the MCMs are loaded to form a double hexamer (80). Phosphorylation (79), SUMOylation (81), and ubiquitination (82, 83) have been identified to distinct subunits of MCMs to regulate their recruitment, activation and disassembly. Interestingly, MCM3, 6, 7 are O-GlcNAcylated, with the alteration pattern of Mcm7 different from Mcm3 and 6 (53). The significance of this modification is still up in the air.

### O-GlcNAcylated proteins in mitosis

All four core histones, H2A, H2B, H3, and H4 are found to be O-GlcNAcylated (63). By centrifugal elutriation, histone O-GlcNAcylation is found to be high in the G1 phase, decreases during the S phase and elevates during S/G2 transition (63). Glycosylated H3, specifically, is higher in interphase than mitosis (56). Histone H3 Ser10 phosphorylation (H3pS10), a landmark of mitosis, is interconnected with O-GlcNAcylation. Mechanistically, H3 is O-GlcNAcylated at T32, and O-GlcNAcylated H3 reduces H3pS10 (56). In general, overproduction of OGT increases H3K9Ac and H3K27me3 levels in mitosis, decreases H3pS10 levels in mitosis and decreases H3R17me2 levels in both asynchronous and mitotic cells (58).

Besides histones, our study about Fizzy-related protein homolog (Cdh1) provides another example of the yin-yang paradigm of O-GlcNAc. Cdh1 is the coactivator of the E3 ligase, anaphase promoting complex (APC), and is phosphorylated at multiple sites (84), with four significant phosphorylation residues at S40, T121, S151, and S163 suppressing the activity of APC (85). O-GlcNAc of Cdh1 has been identified *in vitro* (86). Our assays reveal that O-GlcNAcylation occurs at a peptide harboring S40, and O-GlcNAcylation antagonizes phosphorylation, thus activating APC (51).

The proto-oncoprotein Ewing Sarcoma Breakpoint Region 1 (EWS) binds with both RNA and DNA and localizes to the nucleus, cytosol and cell membranes. Upon adipogenic stimuli EWS is O-GlcNAcylated, which partly promotes its nuclear localization (65). In mitosis, EWS recruits Aurora B and CPC to the spindle midzone (87). In OGA-knockdown cells, O-GlcNAcylation of EWS elevates significantly, resulting in uneven distribution of the spindle mizdone (61).

In an extensive glycoproteomic and phosphoproteomics screen, 141 new O-GlcNAc sites were pinpointed on proteins involved in the spindle apparatus and cytokinesis (50). Many proteins involved in mitosis have also been identified to be O-GlcNAcylated, including the nuclear mitotic apparatus protein 1 (NuMA1), nuclear pore protein 153 (Nup153) and BRCA2-interacting transcriptional repressor EMSY (50). Follow-up investigations reveal that NuMA1 interacts with Galectin-3 in an O-GlcNAcylation-dependent manner (64). Galectin-3 is a small soluble lectin of the Galectin family that localizes to the centrosomes. The newly identified Galectin-3-O-GlcNAc-NuMA1 complex not only localizes NuMA1 to spindle poles, but also is essential for mitotic spindle cohesion (64).

Paradoxically, OGT itself is subject to O-GlcNAcylation (58, 88), which is completely abolished during mitosis, and reappears in G1 (58). It will be an intriguing possibility that O-GlcNAcylation of OGT itself promotes its own stability, so that its abundance declines when the modification drops. Further studies will surely provide more mechanistic insights of O-GlcNAc in mitosis.

### O-GlcNAclated proteins in cytokinesis

Vimentin is an intermediate filament protein, and there have been a few reports on its O-GlcNAcylation. Vimentin is O-GlcNAcylated during M phase, and is also subject to phosphorylation by Cdk1, Plk1, Aurora B, and Rho kinase (21–25, 57). *In vitro* studies suggest that vimentin interacts with GlcNAc-bearing polymers, which promotes phosphorylation of vimentin S71 (66). Cellular assays suggest that vimentin pS71 levels increase when either OGT or OGA is overexpressed (57). Our studies show that when cells are synchronized in cytokinesis, O-GlcNAcylation of vimentin promotes pS71 (26). *OGT* knockdown by siRNA significantly attenuates vimentin pS71, thus forming elongated vimentin bridges during cytokinesis, and impeding daughter cell separation (26). Moreover, Chk1, a kinase pivotal for both DDR and cytokinesis, phosphorylates OGT at S20 specifically during cytokinesis. This phosphorylation is vital for OGT to localize to midbodies and maintain cellular O-GlcNAcylation levels (26). Thus, OGT-S20A mutant cells display vimentin bridge defects due to compromised O-GlcNAcylation levels and consequently quench pS71 of vimentin (26). It will be of keen interest to explore more O-GlcNAcylated proteins in cytokinesis.

## O-GlcNAc in the DDR: a guardian of the genome

Our discussion on cell cycle events will not be complete without DDR. The genomic DNA is subject to many intrinsic or extrinsic lesions, such as double-strand breaks (DSBs), DNA crosslinking damage or DNA alkylation damage. Failure to repair these damages will lead to not only chromosomal abnormalities on the cellular level, but also fatal diseases such as cancer (89). Facing these genomic insults, organisms develop elaborate surveillance mechanisms-DNA damage checkpoints-to ensure genome integrity (90). The term “checkpoint” was coined by the Nobel Laureate, Dr. Leland Hartwell, almost three decades ago to describe the series of concerted responses to deleterious signals, including cell cycle arrest, transcription activation or suppression, and DNA repair. If the damage is beyond repair, then cells might undergo senescence or apoptosis (91).

**Figure 2:**
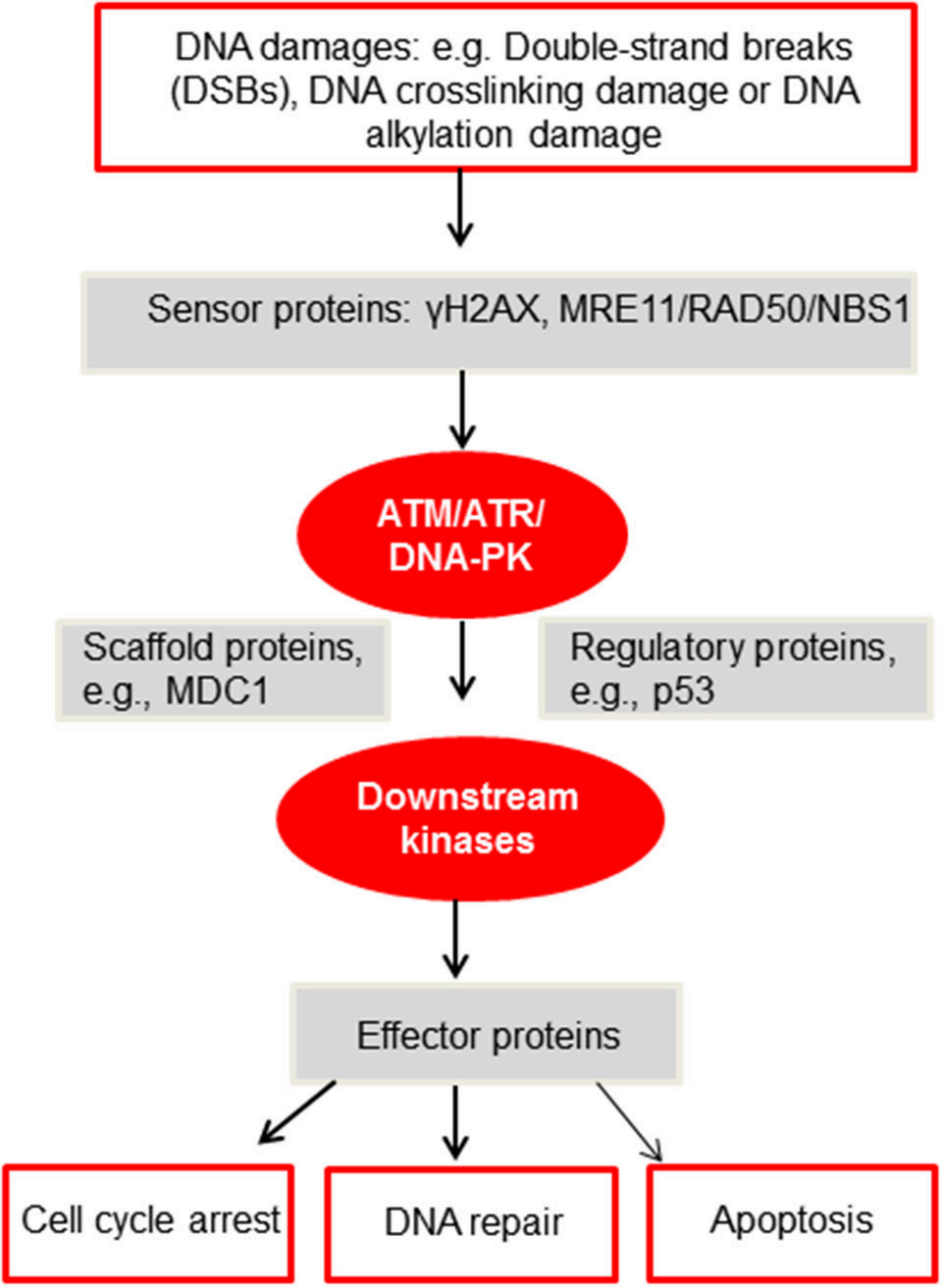
A simplified diagram of the DDR network adapted from Furgason and Bahassi (94). When genomic DNA is faced with various DNA damages, such as but not limited to DSBs, DNA crosslinking damage or alkylation damage, sensor proteins such as γH2AX and the MRE11/RAD50/NBS1 (MRN) complex will then recruit and activate the PIKK kinases, including ATM, ATR and DNA-PK. These PIKK kinases will then phosphorylate downstream targets, including scaffold proteins, regulatory proteins and downstream kinases. Upon activation, these proteins will subsequently phosphorylate effector proteins to undergo cell cycle arrest, DNA repair or apoptosis. For a detailed mechanistic analysis of the DDR, please refer to Ciccia and Elledge (92).

In-depth investigations have identified an extensive network of proteins as guardians of our genomes to sense, mediate, and repair the damages (92). And these proteins are subject to phosphorylation, mono- or polyubiquitination, or SUMOylation. The very first step in the DDR pathway is the phosphorylation of H2AX (γH2AX) by a group of phosphatidylinositide-3 (PI-3)-like kinases, including ataxia telangiectasia mutated (ATM), ATM, and Rad3 related (ATR) and DNA-dependent protein kinase (DNA-PK) (92). Then γH2AX is recognized by checkpoint mediator proteins, such as mediator of DNA damage checkpoint protein 1 (MDC1). MDC1, along with other mediators, is phosphorylated by ATM, and the DDR signal is propagated downstream to transducer kinases, such as Chk1 and checkpoint kinase 2 (Chk2) (92), to execute homologous recombination (HR) or non-homologous end joining (93) and other repair pathways (92) (Figure 2).

As O-GlcNAcylation is deemed as a stress signal, its effects on DNA damage have been of intense interest. As early as 2004, global O-GlcNAcylation levels were investigated after cells were irradiated by the ultraviolet (UV)-B light for 90 s, then recovered for 8 h. It was discovered that O-GlcNAc increased dose-dependently on the UV radiation durations (95). Then in 2015, a comprehensive proteomic study using OGT-null MEFs demonstrated that 232 phosphosites were upregulated compared with OGT wild-type cells (20), among which were ATM pS1987 and Chk1 pS317. Recently, O-GlcNAc was caught at the scene of crime. DNA damages increase O-GlcNAc levels at the damage sites, where both OGT and OGA are recruited (96). OGT abrogation reduces cell viability during DDR (96).

In spite of the significance of DDR, a search of “O-GlcNAc, DNA damage response” in the PubMed only yielded 12 publications. The 2015 quantitative phosphoproteomics study (20) thus provides us a timely route map to explore this uncharted territory. Herein we will review the O-GlcNAcylated DDR proteins, and more importantly, its underlying molecular underpinnings (Table 3).

**Table 3:** O-GlcNAcylated proteins during the DNA damage response.

**Protein name**	**Molecular mechanisms**	**References**
Histone H2AX (H2AX)	H2A is O-GlcNAcylated at T101 at basal levels, and at S139 upon DDR.	(96)
Histone 2B (H2B)	DSBs induce O-GlcNAcylation at S112 of H2B, leading to its binding with Nijmegen breakage syndrome 1 (NBS1), mediating focus formation of NBS1 and subsequent homologous recombination (HR) and nonhomologous end-joining (NHEJ)	(97)
Histone 2A (H2A)	Camptothecin (CPT) or Etoposide (ETP) induces O-GlcNAcylation at S40 of H2A, which coIPs with acetylated H2AZ and γH2AX. O-GlcNAcylated H2AS40 localizes to DNA damage sites. Its aberration prevents recruitment of DNA-PKcs and Rad51.	(98, 99)
Ataxia-telangiectasia mutated (ATM)	In HeLa and primary neuron cells, ATM interacts with OGT and is O-GlcNAcylated. O-GlcNAcylation enhances X-ray induced ATM activation at S1981.	(100)
	In mouse embryonic fibroblasts (MEFs), OGT deletion upregulates ATM activation at S1987.	(20)
DNA-dependent protein kinase (DNA-PK)	DNA-PK is O-GlcNAcylated, and this modification increases upon ER stress, but not upon oxidative stress, osmotic stress, or double-strand breaks (DSBs).	(101)
Mediator of DNA damage checkpoint protein 1 (MDC1),	O-GlcNAcylation of H2AX suppresses its expansion on chromatin during DDR. MDC1 is O-GlcNAcylated upon DDR, which antagonizes its phosphorylation.	(96)
Polη	O-GlcNAcylation at T457 of Polη promotes CRL4^CDT2^-dependent Polη polyubiquitination at K462 and subsequent p97-dependent removal from replication forks, ensuring translesion DNA synthesis (TLS)	(32)

### O-GlcNAcylation of histones

Histone modification is integral to epigenetics, and OGT has been central to epigenetics (102). Here we will only cover histones involved in DDR, including H2AX, H2AS40, and H2B.

Histone H2A has many variants (103) and γH2AX is vital to DDR. H2AX is O-GlcNAcylated at T101 at basal levels and at S139 upon DDR, which restricts the expansion of damage signals, thus limiting DDR (96).

Viviparous animals have either Ser40 (S40) or Ala40 (A40) types of H2A, while non-mammals only have A40 (98, 99). Specific to viviparous organisms, Camptothecin (CPT) or Etoposide (ETP) (both inhibitors for Topoisomerase) induce O-GlcNAcylation at S40 of H2A (98, 99). This modification interacts with γH2AX, recruits DNA-PK and the recombinase Rad51 to the damage sites, and facilitates DDR (98, 99).

Besides H2AX and H2AS40, H2B is O-GlcNAcylated at S112 upon DSBs (97). Then the O-GlcNAcylated H2B binds with the Nijmegen breakage syndrome 1 (NBS1) protein, promoting its recruitment to DSBs and the following repairing events.

### O-GlcNAc of kinases and scaffolds

Among the DDR proteins, the ATM, DNA-PK kinases, and the scaffold protein MDC1 have been reported to be O-GlcNAcylated. O-GlcNAc of DNA-PK is elevated upon ER stress, but not during oxidative, osmotic or DSB stresses (101). The mediator MDC1 is also O-GlcNAcylated, which counteracts ATM-dependent phosphorylation (96).

Reports about ATM have been controversial. Miura et al. (100) reported that in HeLa cells and primary cultured neurons ATM is O-GlcNAcylated (with CTD110.6) and endogenous ATM coIPs with OGT. O-GlcNAc of ATM enhances ATM activation at S1981 upon X-ray (100), thus O-GlcNAc plays a positive role. Meanwhile, Zhong et al. (20) failed to detect O-GlcNAc of ATM in MEFs. OGT deletion upregulates the activation phosphorylation of ATM at S1987 (the mouse equivalent of human S1981) (20), thus OGT plays a negative role. The discrepancy might lie in different species. Phosphorylation of S1981 has been a hallmark of ATM activation in humans, but mutation of S1987 to Ala does not impede ATM function in mice (104, 105). More in-depth studies are needed to solve the mystery.

The exact modification sites of these proteins were not investigated, probably because ATM, DNA-PK, and MDC1 are all large proteins. Fragmentation studies and precise site mapping will be needed in the future. And whether there are other distinctions between mice and humans, as far as O-GlcNAc is concerned, is at anyone's wild guess.

### OGT in DNA translesion synthesis (TLS)

TLS utilizes a flurry of distinct and specialized DNA polymerases to replicate damaged regions of DNA (106). Polη is specifically involved in replicative bypass of cyclobutane pyrimidine dimers (CPDs) induced by UV, as well as damages induced by cisplatin (107). It is error prone, with an error rate of 10^−2^-10^−3^, thus it is vital to timely remove Polη after DDR.

A peptide of Polη was O-GlcNAcylated *in vitro* (86) and T457 was identified to be O-GlcNAcylated upon DDR in cultured cells (32). This modification promotes polyubiquitination at K462 and subsequent removal of Polη from replication forks. Moreover, O-GlcNAcylated Polη is essential for cisplatin-induced DDR. The deficient T457A mutant enhances UV-induced mutagenesis and also increases cellular sensitivity to cisplatin (32).

Collectively, the reports of OGT in DDR are emerging. From the current few investigations, O-GlcNAc seems to play an overall positive role: OGT is recruited to damaged sites, where it helps recruit NBS1; it antagonizes phosphorylated H2AX and MDC1 to restrict damage signal expansion; it promotes error-prone Polη to dissociate from chromatin after TLS to bypass UV-induced CPDs. Considering that DDR utilizes distinct pathways in response to each and every DNA damage type, we need more time and energy to elucidate how O-GlcNAc fine-tunes the DDR process.

## Conclusions

In less than two score years, great strides have been made in the field of O-GlcNAcylation. We have witnessed really exciting things in glycobiology, and its utilization in medicine and clinical practice. As we have realized, most bedside applications will depend on our understandings of underlying molecular mechanisms to the ultimate level of resolution. Achieving that will not only enable us to comprehend various biological events, but also bestow upon us rich information as well as rich opportunities to be exploited. As we begin to elucidate the molecular basis of O-GlcNAc and its interaction, we are going to be able to leap profoundly forward into our future.

## Author contributions

All authors listed have made a substantial, direct and intellectual contribution to the work, and approved it for publication.

### Conflict of interest statement

The authors declare that the research was conducted in the absence of any commercial or financial relationships that could be construed as a potential conflict of interest.

## Abbreviations

(O-GlcNAc): O-linked N-acetylglucosamine
(OGT): O-GlcNAc transferase
OGA: O-GlcNAcase
DDR: DNA damage response
TLS: DNA translesion synthesis
ETD: electron transfer dissociation
MS: mass spectrometry
HCD: higher energy collisional dissociation
PTM: post-translational modification
IP: immunoprecipitate
IB: immunoblot
UV: ultraviolet
MEF: mouse embryonic fibroblast
DSB: double-strand break
ATM: ataxia telangiectasia mutated
ATR: ATM and Rad3 related
DNA-PK: and DNA-dependent protein kinase
MDC1: mediator of DNA damage checkpoint protein 1.
